# Secretion of Iron(III)-Reducing Metabolites during Protein Acquisition by the Ectomycorrhizal Fungus *Paxillus involutus*

**DOI:** 10.3390/microorganisms9010035

**Published:** 2020-12-24

**Authors:** Firoz Shah, Markus Gressler, Susan Nehzati, Michiel Op De Beeck, Luigi Gentile, Dirk Hoffmeister, Per Persson, Anders Tunlid

**Affiliations:** 1 Microbial Ecology Group, Department of Biology, Lund University, 223 62 Lund, Sweden; firoz.shah@slu.se (F.S.); susan.nehzati@maxiv.lu.se (S.N.); michiel.op_de_beeck@biol.lu.se (M.O.D.B.); luigi.gentile@uniba.it (L.G.); 2 Department of Pharmaceutical Microbiology at the Hans Knöll Institute, Friedrich-Schiller-Universität, 07747 Jena, Germany; Markus.Gressler@hki-jena.de (M.G.); Dirk.Hoffmeister@hki-jena.de (D.H.); 3 MAX IV Laboratory, Lund University, 221 00 Lund, Sweden; 4 Centre for Environmental and Climate Research (CEC), Lund University, 223 62 Lund, Sweden; per.persson@cec.lu.se

**Keywords:** ectomycorrhizal fungi, Fenton reaction, involutin, iron reduction, secondary metabolites

## Abstract

The ectomycorrhizal fungus *Paxillus involutus* decomposes proteins using a two-step mechanism, including oxidation and proteolysis. Oxidation involves the action of extracellular hydroxyl radicals (•OH) generated by the Fenton reaction. This reaction requires the presence of iron(II). Here, we monitored the speciation of extracellular iron and the secretion of iron(III)-reducing metabolites during the decomposition of proteins by *P. involutus*. X-ray absorption spectroscopy showed that extracellular iron was mainly present as solid iron(III) phosphates and oxides. Within 1 to 2 days, these compounds were reductively dissolved, and iron(II) complexes were formed, which remained in the medium throughout the incubation. HPLC and mass spectrometry detected five extracellular iron(III)-reducing metabolites. Four of them were also secreted when the fungus grew on a medium containing ammonium as the sole nitrogen source. NMR identified the unique iron(III)-reductant as the diarylcyclopentenone involutin. Involutin was produced from day 2, just before the elevated •OH production, preceding the oxidation of BSA. The other, not yet fully characterized iron(III)-reductants likely participate in the rapid reduction and dissolution of solid iron(III) complexes observed on day one. The production of these metabolites is induced by other environmental cues than for involutin, suggesting that they play a role beyond the Fenton chemistry associated with protein oxidation.

## 1. Introduction

A large part of the nitrogen (N) present in forest soils is found in organic forms, primarily as proteins and peptides. These molecules are typically complexed with polyphenols, polysaccharides, lignin residues, and other degradation products of litter material that constitute soil organic matter (SOM) [1,2]. Moreover, a major fraction of soil organic N is associated with mineral particles [3]. Forest trees have a limited capacity to access the soil organic N by themselves [4]. Organic and mineral N-containing complexes need to be decomposed by microorganisms before they become available to plants [5]. A long-standing hypothesis is that ectomycorrhizal (ECM) fungal symbionts play a key role in this process [6]. However, considering the fact that ECM fungi have lost many of the genes encoding plant litter degrading enzymes that are present in saprotrophic fungi [7,8], the extent to which ECM fungi decompose SOM and mobilize organic N, and the mechanisms by which they do so, are still unclear [9].

Laboratory experiments have shown that a number of ECM fungi have at least some capacity to decompose SOM by means of oxidative mechanisms [10]. Using time-series spectroscopy and transcriptomics, we recently demonstrated that the oxidation of organic matter by ECM fungi is linked to the liberation of organic N from SOM and that those events are controlled by the availability of C (i.e., glucose) and N [11]. Those experiments were conducted with *Paxillus involutus* and *Laccaria bicolor*, representing two independently evolved ECM lineages [7]. In both species, oxidation was initiated when the readily available N source, ammonium, was depleted. Following oxidation, organic N sources, including amide-N, were utilized. Despite those similarities, the decomposition mechanisms, including the type of genes involved and the patterns of their expression, differ markedly between the two species. The data suggest that SOM decomposition by *P. involutus* is a two-step mechanism of oxidation and hydrolysis, whereas *L. bicolor* uses a one-step mechanism that involves a combined activity of oxidative and hydrolytic enzymes [11].

The oxidation of SOM by *P. involutus* is due to the action of hydroxyl radicals (•OH) generated by the nonenzymatic Fenton reaction (Fe^2+^ + H_2_O_2_ + H^+^→Fe^3+^ + •OH + H_2_O), similar to the mechanism used by brown-rot fungi to decay wood [12]. In brown rot fungi, the Fenton reaction reagents are generated by secreted metabolites that drive the reduction of Fe^3+^ to Fe^2+^ and O_2_ to H_2_O_2_ [13,14]. Like brown rot fungi, *P. involutus* secretes iron(III)-reducing metabolites during organic matter decomposition [15]. Mass spectrometry and nuclear magnetic resonance (NMR) spectroscopy identified the major compound secreted by *P. involutus* during oxidative decomposition of a maize compost extract as the diarylcyclopentenone involutin. The metabolite was not detected when the fungus was grown on a mineral medium containing no organic nutrient sources [15]. Moreover, the key step in the biosynthetic pathway for involutin has been characterized. The metabolic precursor to involutin has been identified as the terphenylquinone atromentin, which is synthesized by three redundant quinone synthetases [16]. Atromentin is a precursor to a large number of secondary metabolites in basidiomycetes [17].

Recently, we have characterized the nutritional signals that induce extracellular •OH production in *P. involutus* in more detail. The fungus was grown on mineral media containing different organic compounds representing major components of SOM [18]. Though *P. involutus* produced low but steady amounts of •OH in all media, high levels of radicals were only produced in growth medium containing protein (bovine serum albumin, BSA) and ammonium. In agreement with the time-course experiments with complex SOM [11], enhanced •OH production started after the ammonium in the growth medium was depleted. Extracellular proteolytic activity was initiated shortly after the onset of elevated •OH production. These experiments support the view that the production of •OH and extracellular proteolytic enzymes are regulated by similar nutritional and cellular signals and that they operate in a sequence, with oxidation acting before proteolysis.

A notable observation in the above experiments is that extracellular iron(III) is reduced to iron(II) by *P. involutus* when grown on a range of different media, including those that do not induce the extracellular •OH production associated with decomposition of proteins [18]. Moreover, when grown on media with BSA and ammonium, the iron(II) concentration immediately starts to increase in the medium, much earlier than enhanced •OH production and protein oxidation are recorded [18]. Collectively, these data suggest that a high iron(II) concentration is a prerequisite for, but does not necessarily result in, an induction of •OH production. Accordingly, iron(III)-reducing metabolites produced by *P. involutus* may not only be involved in generating the Fenton chemistry reactants but also in other processes. To gain insights into such extracellular redox processes, the speciation of iron and its binding environment during oxidative decomposition of protein by *P. involutus* was followed using X-ray absorption spectroscopy (XAS). In parallel, the production and characterization of iron(III)-reducing metabolites was followed using HPLC and mass spectrometry. In particular, we asked the following two questions: (1) What specific forms of extracellular iron species occur when *P. involutus* is grown on BSA-ammonium medium and what iron(III)-reductants are secreted? (2) Are any specific iron(III)-reducing metabolite(s) secreted that can be linked to an enhanced production of •OH, which occurs when the fungus switches from ammonium to proteins as the N source? The time-course experiments revealed large and rapid changes in the forms of extracellular iron during growth on a medium containing both BSA and ammonium. Five low molecular weight, extracellular iron(III)-reductants were identified, of which four were also secreted when the fungus was grown on a medium containing ammonium as the sole N source. One of these metabolites, identified as involutin, was only secreted in the protein-containing medium, supporting the hypothesis that this metabolite has a key role in the Fenton chemistry associated with protein degradation in *P. involutus*. The other, not yet fully characterized, iron(III)-reducing metabolites may be involved in the reductive dissolution of solid iron(III) oxide and iron(III) phosphate complexes that are present in the medium at the onset of the experiments.

## 2. Materials and Methods

### 2.1. Fungal Strain and Culture Conditions

Cultures of *P. involutus* (Batsch) Fr. (strain ATCC 200175) were maintained on modified Fries medium containing 3.74 mM NH_4_Cl, 0.41 mM MgSO_4_·7H_2_O, 0.22 mM KH_2_PO_4_, 0.18 mM CaCl_2_·2H_2_O, 0.34 mM NaCl, 1.34 mM KCl, 0.24 mM H_3_BO_3_, 20 µM ZnSO_4_·7H_2_O, 5.01 µM CuSO_4_·5H_2_O, 50.29 µM MnSO_4_·H_2_O, 0.16 µM (NH_4_)_6_Mo_7_O_24_·7H_2_O, 73.99 µM FeCl_3·_6H_2_O, 33.30 mM d-glucose, 55.51 µM myo-inositol, 0.30 µM thiamine-HCl, 0.10 µM biotin, 0.59 µM pyridoxine, 0.27 µM riboflavin, 0.82 µM nicotinamide, 0.73 µM p-aminobenzoic acid, 0.46 µM Ca-pantothenate, and 1% (*w*/*v*) agar. The pH was adjusted to 4.8. Cultures were grown at 22 °C in the dark. For the experiments, the fungus was cultivated in Petri dishes on a monolayer of glass beads immersed in liquid Fries medium, and the experiments were conducted as previously reported [18]. In short, after 9 days of incubation, the culture medium was removed, the glass beads and the mycelium were washed with 10 mL of sterile Milli-Q (MQ) water, and 10 mL of Fries medium without N was added to induce an N-deprived mycelium [19]. After 24 h, the mycelium was washed again with MQ water, and 10 mL of Fries medium supplemented with 331.5 mg L^−1^ BSA was added. In addition to these “BSA” samples, the fungus was grown on Fries medium without BSA. These latter samples were designated as “BSA-free”. In all experiments, growth media were filter sterilized.

### 2.2. Iron(III)-Reducing Activities and Fe^2+^ Concentrations

The iron(III)-reducing activities of fungal metabolites/culture extracts were analyzed using the ferrozine assay as previously described [15]. The concentrations of Fe^2+^ in the culture filtrates were also determined using the ferrozine assay without adding FeCl_3·_6H_2_O to the reaction mixture [18]. In both assays, the formation of the Fe^2+^-ferrozine complex was measured after 5 min of incubation (room temperature) at λ = 562 nm using an Ultrospec 3000 UV-visible light spectrophotometer (GE Healthcare, Chicago, IL, USA). Iron(III)-reducing activities of fungal metabolites/culture extracts were calculated as the difference in absorbance at 562 nm of Fe^2+^-ferrozine complexes formed in ferrozine assays with FeCl_3_·6H_2_O and without FeCl_3_·6H_2_O added. A standard curve was constructed using FeSO_4_·7H_2_O.

### 2.3. Determination of •OH Production

The extracellular production of •OH by *P. involutus* cultures was followed over time using 100 µM coumarin added to growth media [18]. When coumarin is oxidized by •OH, one of the reaction products is the fluorescent molecule umbelliferone. Umbelliferone concentrations were estimated by measuring the fluorescence intensity (λ_ex_ = 325 nm, λ_em_ = 455 nm) in culture filtrates using an LS50B fluorescence spectrophotometer (PerkinElmer, Waltham, MA, USA). Before the fluorescence measurements, culture filtrates were acidified to pH 2, as previously described [18].

### 2.4. X-ray Absorption Spectroscopy (XAS)

Culture filtrates from the BSA medium were collected daily from day 0 to 7. The filtrates were lyophilized to dryness and packed into a metal sample holder sealed with Kapton tape. Fe K-edge spectra were collected at beamline 4–1 at the Stanford Synchrotron Radiation Lightsource (SSRL) with the SPEAR3 storage ring maintaining 500 mA at 3.0 GeV. The beamline was equipped with a liquid-nitrogen cooled Si(220) double-crystal monochromator, and the incident X-ray intensity was monitored with a nitrogen-filled ion chamber. Internal energy calibration was simultaneously monitored using the spectra of Fe foil recorded with a nitrogen-filled ion chamber placed behind the sample. The sample holder was placed in a liquid-nitrogen-cooled cryostat at 45° to the incident X-ray beam, and fluorescence was measured using a passivated-implanted planar silicon (PIPS) detector. Unwanted fluorescence and scattering contributions to the signal were minimized using Soller slits and an Mn filter (6 µx).

The XAS spectra were analyzed using the VIPER program [20]. Individual XAS scans were energy calibrated using the lowest-energy inflection point of Fe foil, assumed to be 7111.08 eV. The spectra were averaged, normalized, and background removal was accomplished using a Bayesian smoothing spline function. Extended X-ray absorption fine structure (EXAFS) oscillations were fit using theoretical phase and amplitude functions from models of amorphous FePO_4_ [21] and goethite [22] calculated using the software FEFF7 [23]. The amplitude reduction factor $\left(S_{0}^{2}\right)$ was set to 1.00. The threshold energy shift (ΔE_0_) was allowed to float, yet assumed to be identical for all paths. The Debye-Waller factors (σ^2^) for the Fe-P path was fixed to 0.0085, determined by Sundman et al. 2015 [21] and the Fe-Fe path was fixed to 0.0100, following Sundman et al. 2014 [24]. The integrated intensity and centroid energy position of the pre-edge peak were determined following the procedure described by Wilke et al. 2001 [25].

### 2.5. Identification of Extracellular Secondary Metabolites

Culture filtrates from 7-day old cultures of *P. involutus* grown on BSA and BSA-free media were extracted with ethyl acetate [15]. Equal volumes of culture filtrates and ethyl acetate were mixed with 1 M HCl (ratio 5:5:1) and vortexed. The ethyl acetate phase was recovered and dried under a stream of N_2_. The dried ethyl acetate extracts were dissolved in methanol. Dissolved molecules were separated using high-performance liquid chromatography (HPLC) and analyzed using liquid chromatography-mass spectrometry (LC–MS). HPLC was performed on a Dionex Ultimate 3000 (Thermo Fisher Scientific, Waltham, MA, USA) instrument coupled with a guard cartridge and a Kinetex C_18_ column (150 × 4.6 mm, 2.6 µm particle size; Phenomenex, Værløse, Denmark). An elution gradient consisting of 0.1% (*v*/*v*) formic acid in HPLC-grade water (solvent A) and acetonitrile (solvent B) was applied at a flow rate of 0.36 mL min^−1^, using a gradient elution (initial hold, 5 min at 5% solvent B, followed by a linear increase over 35 min to 40% B, then a linear increase over 5 min to 90% B, hold for another 5 min, then return to the initial condition of 5% B within 5 min). Chromatograms were recorded at 210, 254 and 280 nm, using data from a variable wavelength UV detector (Dionex Ultimate 3000). Fractions were collected based on peak retention time, dried under a stream of N_2_, and analyzed for their iron(III)-reducing activity.

The LC/MS analysis was conducted with an Agilent UHPLC-MS 1290 Infinity II instrument with a 6130 quadrupole MS detector (Agilent Technologies, Santa Clara, CA, USA) equipped with the same column used for HPLC. A shorter elution gradient consisting of the above HPLC solvents was applied at a flow rate of 1 mL min^−1^ with the following gradient configuration: initial hold, 2 min with 5% B, followed by a linear increase over 20 min to 40% B, then increase to 90% B over 5 min, hold for 5 min, then a decrease to 5% B over 5 min). Electrospray ionization (ESI) data were acquired in both negative and positive mode with an ion mass range from *m*/*z* 250 to 1000. UV/Vis spectra were recorded from λ = 210 to 400 nm. Identified peaks at λ = 254 nm were then isolated from the BSA culture extract based on peak retention time on an Agilent 1200 integrated HPLC system with fraction collector, using the same column and elution gradient (Agilent Technologies, Santa Clara, CA, USA). The isolated compounds were analyzed by high-resolution electrospray ionization-mass spectrometry (HRESIMS) on a Q Exactive Orbitrap instrument (Thermo Fisher Scientific). Ions were recorded in negative and positive modes. The instrument was equipped with a Betasil C_18_ column (2.1 × 150 mm; 3 µm particle size; Thermo Fisher Scientific). The flow rate was 0.2 mL min^−1^. Solvent A was 0.1% (*v*/*v*) formic acid in water, solvent B was 0.1% (*v*/*v*) formic acid in acetonitrile, and the following gradient was used: an initial hold at 5% B for 1 min, then a linear increase to 98% B within 15 min, followed by a hold at 98% B for 3 min.

### 2.6. Production of Extracellular Iron(III)-Reducing Metabolites

Culture filtrates from the BSA and BSA-free growth conditions were collected on incubation days 0, 1, 2, 4 and 7, extracted with ethyl acetate and analyzed using a Dionex Ultimate 3000 HPLC (Thermo Fisher Scientific) instrument and the conditions described above. The area under the peak detected at λ = 254 nm representing the iron(III)-reducing fungal metabolite identified using mass spectrometry was calculated using the Chromeleon ver. 7.2 software. In the same way, the relative amounts of the iron(III)-reducing metabolites were estimated in filtrates collected from cultures before and after the N-starvation treatment, i.e., before adding the BSA and BSA-free media.

### 2.7. NMR Spectroscopy

To produce sufficient amounts of involutin for NMR analysis, *P. involutus* was grown for 2 months on liquid malt extract (2%, *w*/*v*) in Petri dishes containing glass beads as described above. Metabolites were extracted from the culture filtrates and purified by HPLC (Dionex Ultimate 3000, Thermo Fisher Scientific) using a Kinetex C_18_ column and conditions as described above. The mass spectrum of the peak putatively assigned to involutin was recorded in negative mode (ESI) using an LTQx Orbitrap (Thermo Fisher Scientific). The ^1^H NMR spectrum of the collected fraction was recorded on a Bruker Avance III HD 500 MHz spectrometer (Bruker, Billerica, Massachusetts, USA), equipped with a 5 mm broadband probe, optimized for ^1^H observation. All spectra were recorded with the same settings, i.e., an acquisition time of 2 s, a relaxation delay of 6 s, and 512 scans. The involutin sample obtained from the HPLC purification was dissolved in methanol-d_4_ (99.8%; Deutero GmbH, Kastellaun, Germany), and chemical shifts in standard ^1^H experiments were referenced to the resonances of residual protons in deuterated methanol.

## 3. Results

### 3.1. OH Production and Iron(III) Reduction

**Figure 1:**
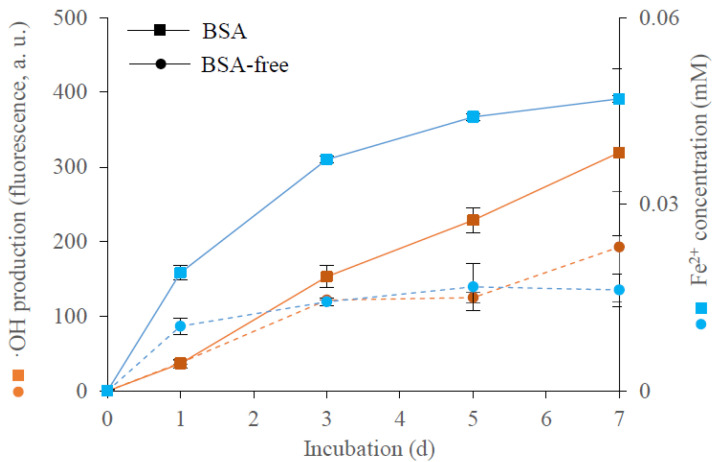
Production of extracellular hydroxyl radicals (•OH) and reduction of Fe^3+^ by *P. involutus* when grown for 7 days on BSA-containing and BSA-free media. Reduction of Fe^3+^ was estimated as the concentration of Fe^2+^ in the culture filtrate. Bars indicate ± standard deviations (*n* = 3).

In agreement with data from previous experiments [18], the presence of BSA in the growth medium was associated with induced •OH production (Figure 1). Enhanced •OH production was observed from day 3 of the incubation period, which coincides with the depletion of ammonium in the growth medium [18]. In the BSA growth medium, iron(III) was reduced to iron(II), and the concentration of Fe^2+^ increased during incubation (Figure 1). Reduction of iron(III) to Fe^2+^ was also observed when the fungus was grown on the BSA-free medium. The levels of Fe^2+^ were higher in the BSA than in the BSA-free medium.

### 3.2. Fe Speciation in the Protein Growth Medium

XAS spectroscopy was used to examine the speciation of iron in the BSA medium of *P. involutus*. The Fe K-edge XAS was analyzed in the medium before inoculating the fungus (day 0), and in the medium collected after 1 to 7 days of incubation. The pre-edge peak, X-ray absorption near-edge structure (XANES), and first-derivative spectra of the XANES region are shown in Supplementary Figure S1. This region of XAS spectra reveals information on the coordination environment and oxidation states of iron [25]. Results collectively showed a considerable change of Fe speciation in the culture media between 24 and 48 h after starting the incubation, which can be seen in the change of shape in the XANES spectra and shifting of spectra to lower energies.

**Figure 2:**
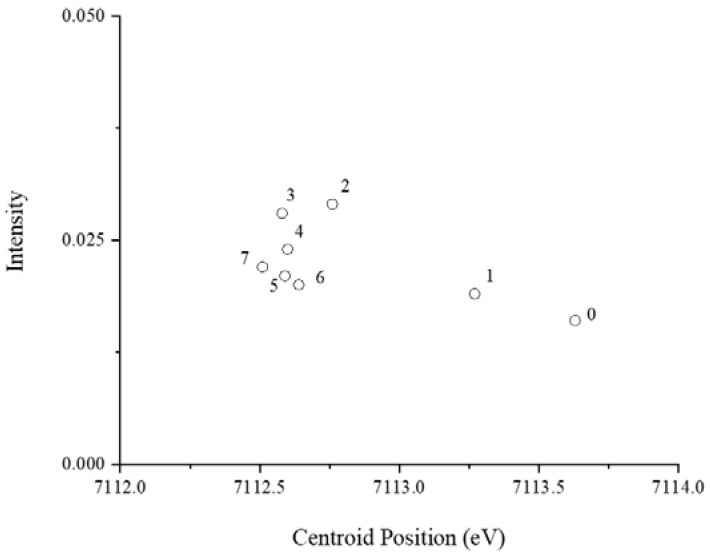
Pre-edge peak centroid energies and integrated pre-edge intensities obtained from the analysis of the X-ray absorption near-edge structure (XANES) region. Samples were collected daily (from day 0 to day 7) from culture filtrates of *P. involutus* growing on BSA-containing medium.

The first-derivative of XANES spectra (Supplementary Figure S1C) indicated a partial shift to lower energy within the first 24 h of incubation. The spectra of the culture media incubated for 48 or more hours show more or less identical coordination environments in the time-series. The pre-edge peak of the time-series was further examined by curve-fitting analysis to aid in distinguishing the coordination and oxidation states of Fe. The centroid positions of Fe compounds are typically 7112.1 eV for Fe(II) and 7113.5 eV for Fe(III), with higher X-ray absorption intensities measured with four-coordinate compounds when compared to six-coordinate compounds [25]. The trend observed in the culture filtrates is illustrated in Figure 2. The initial pre-edge peak position representing day 0 of fresh medium at 7113.63 eV is indicative mostly of iron(III) compounds. As the medium is incubated for longer periods of time, the pre-edge peak position shifts to lower energies, concluding at 7112.51 eV in the culture filtrate, representing day 7. The intensity of the pre-edge peaks through the series increases modestly, suggesting a transformation of Fe coordination from six to four.

**Figure 3:**
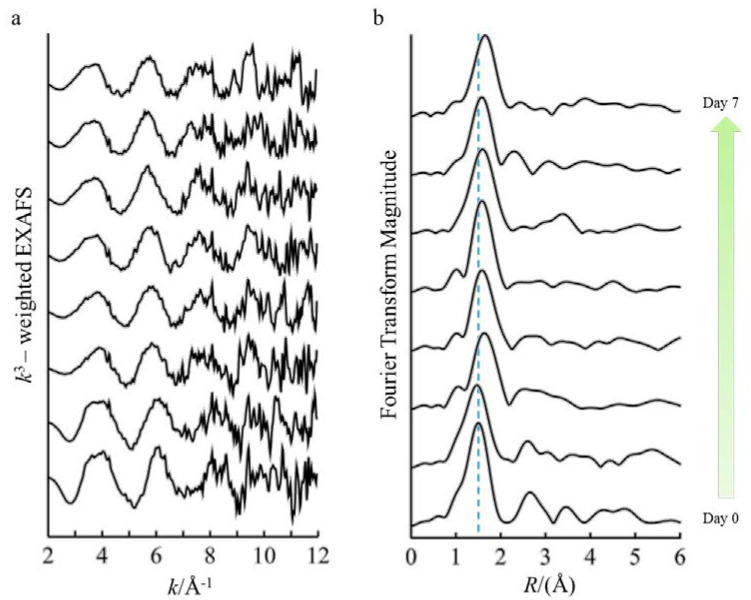
Fe K-edge EXAFS spectra (**a**) and corresponding Fourier transforms (**b**). Samples were collected daily (from day 0 to day 7) from culture filtrates of *P. involutus* growing on BSA-containing medium. The blue dashed line serves to identify changes in the peak positions.

Analysis of the EXAFS spectra revealed results that are consistent with trends observed in the analysis of the XANES region (Figure 3 and Table 1). Modeling of the EXAFS spectra representing day 0 and following 24 h of incubation show first-shell contributions of Fe-O paths at bond distances of 1.99 Å and coordination numbers (CN) 5.9 and 5.6, respectively. After 48 h, the best fit for the first-shell scattering path (Fe-O) comprised of CNs ranging from 3.2 to 4.6 (average CN = 3.9) with noticeably longer corresponding bond-distances varying from 2.08 to 2.12 Å. Modeling of the spectra within the first 24 h also found second and third-shell contributions, but these were not discernible in the spectra after 48 h of incubation. Second-shell fitting for 0 and 24 h of incubation consisted of Fe-P backscattering at distances of 3.19 and 3.15 Å with corresponding CNs of 3.5 and 3.9. The contribution of a third-shell Fe-Fe path was modeled with scattering distances of 3.11 and 3.10 Å and corresponding CNs of 2.8 and 3.9, for 0 and 24 h incubation, respectively. Overall, the EXAFS fitting results together with the XANES analysis clearly reveal that the coordination of Fe transforms from six-coordinated iron(III) phosphate with some contribution from iron(III) oxides to four-coordinated iron(II) complexes as iron is reduced in the growth media of *P. involutus*.

### 3.3. Characterization of Iron(III)-Reducing Metabolites

**Figure 4:**
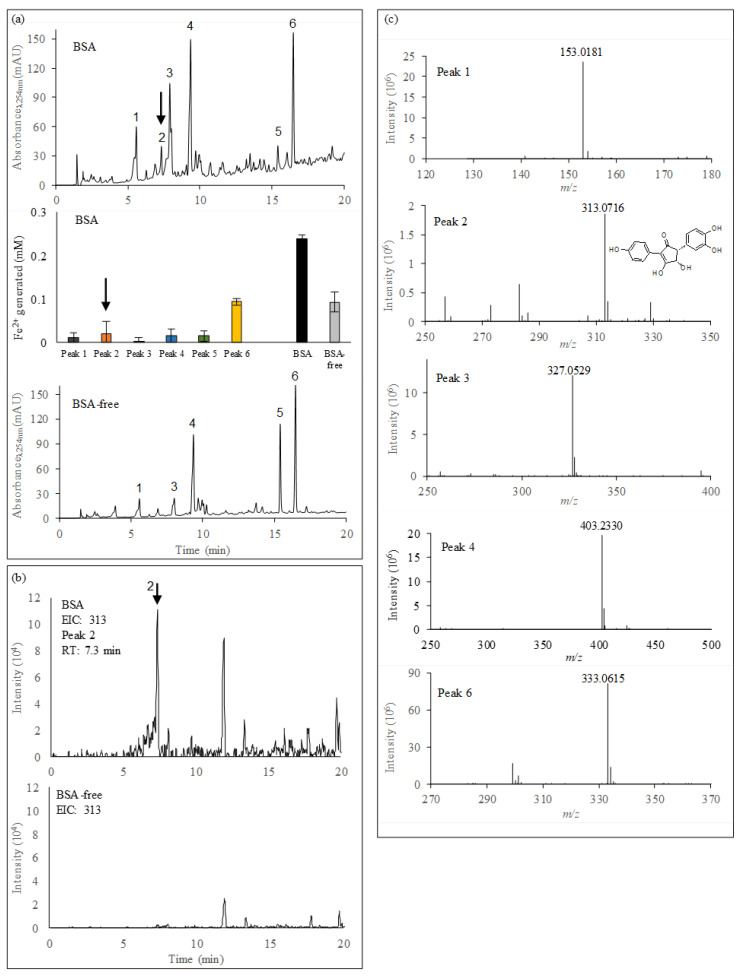
Characterization of iron(III)-reducing metabolites secreted by *P. involutus* when grown on BSA or BSA-free media for 7 days. (**a**) Top panel: HPLC chromatogram of metabolites extracted by ethyl acetate from the BSA culture filtrates. Middle panel: iron(III)-reducing the activity of peaks 1 to 6 recovered from the BSA culture filtrates. Bars indicate ± standard deviations (*n* = 3). Lower panel: HPLC chromatogram of metabolites extracted from the BSA-free culture filtrates. Peak 2 (arrow) was unique to the BSA culture filtrate and was identified as involutin. (**b**) LC-MS profiling (negative ion mode) of the extracts from the BSA-containing (top) and BSA-free (bottom) culture filtrates. The ion chromatogram (EIC) of *m/z* 313 is shown, which corresponds to the molecular ion of involutin (only detected in the BSA culture filtrates (arrow)). (**c**) HRESIMS spectra (negative mode) of the HPLC identified peaks with iron(III)-reducing activity (c.f. (**a**)). Peak 5 was present in the incubation medium lacking BSA and was not analyzed.

HPLC-UV analysis of the ethyl acetate extract and subsequent ferrozine assays of the 7-day old culture filtrate of *P. involutus* grown on media containing BSA revealed the presence of six compounds (peaks 1 to 6) with iron(III)-reducing activities (Figure 4a). Peak 2 was only detected in the extracts from the BSA culture filtrate, while the other peaks were observed in both the BSA and BSA-free culture filtrates. Peak 5 was identified as a component of the mineral nutrient medium and was not analyzed further (Supplementary Figure S2).

LC-MS analysis of the same culture extracts showed peaks corresponding to the iron(III)-reducing compounds identified using UV detection. Mass spectra were recorded in the negative mode. Mass selective detector (MSD) signals at *m*/*z* 313, which corresponds to the molecular ions of involutin ([M-]^−^) [15], identified a peak in the extracts from the BSA culture filtrate, but not in the BSA-free extract (Figure 4b). This peak has a retention time which corresponds to peak 2 in the HPLC-UV analysis. MSD signals in negative and positive modes for the molecular ions corresponding to other atromentin-derived metabolites, including atromentic acid (*m*/*z* 340.0583), gyrocyanin (*m*/*z* 296.0685), gyroporin (*m*/*z* 312.0634), chamonixin (*m*/*z* 298.0841), xerocomic acid (*m*/*z* 356.0532) and variegatic acid (*m*/*z* 372.0481) did not show any significant signals in any of the extracts.

Further characterization of the iron(III)-reductants using HRESIMS identified putative molecular ions ([M-H]^−^) for peak 1: *m*/*z* 153.0181, peak 2: *m*/*z* 313.0716, peak 3: *m*/*z* 327.0529, peak 4: *m*/*z* 403.2330 and peak 6: *m*/*z* 333.0615 (Figure 4c). The predicted elemental compositions of these compounds are shown in Table 2. Apart from involutin (peak 2), none of the predicted compounds showed any significant hits in the Reaxys (Reaxys.com, build 21,227; Elsevier) or the SciFinder (scifinder.cas.org; accessed 2018) databases. A number of secondary metabolites have previously been identified in *P. involutus* and closely related fungal species [25,26]. The fact that the HRESIMS did not reveal any significant match to compounds in the databases suggests that the here detected iron(III)-reductants represent novel, not yet characterized compounds. Moreover, the MS/MS spectra of the isolated iron(III)-reducing metabolites were largely different from each other (Table 2), suggesting that they have diverse molecular structures. However, other methods, including NMR spectroscopy, are needed to elucidate the molecular structures of these novel metabolites.

### 3.4. Identification of Involutin

NMR analysis was used to confirm that the compound present in peak 2 was indeed involutin. Large amounts of the compound present in peak 2 were obtained by growing *P. involutus* on a nutrient-rich medium for a long period of time (2 months). Following extraction with ethyl acetate, material corresponding to peak 2 was isolated using HPLC, and the identity was confirmed using LC-MS (Supplementary Figure S3). The MS analysis showed that the peak contained a single compound with a putative molecular weight of *m*/*z* 313.08. In total, we obtained 2.1 mg per 100 mL medium of the compound denoted as peak 2.

The ^1^H NMR spectrum of the HPLC-purified compound corresponds to involutin (Table 3, Supplementary Figure S4) [15,27]. The ^1^H NMR spectrum of the aromatic region reveals the presence of mainly two tautomeric involutin forms. In particular, Supplementary Figure S4 shows para-disubstituted benzene rings which have two substituents with different electron-withdrawing effects, since a pair of doublets (d), of area ~2 for each of them, centered around 7.74 and 6.76 ppm (^3^J = 8.3 and 8.4 Hz, respectively) were observed. The second tautomeric form (triol) is detected due to another pair of d at 6.71 and 6.58 ppm and one single peak at 6.68 ppm of area ~1 each. The integrated resonances are reported in Supplementary Figure S4C. The MS peak at *m*/*z* 313.08 (Supplementary Figure S3C) denotes involutin, and the identified resonances in the ^1^H NMR spectrum, Table 3, demonstrate the presence of two tautomeric forms of involutin. In the ^1^H NMR spectra, the area of each resonance is relative to the areas of other resonances, in particular, an integer multiple reflecting the overall composition of the molecule. Keto-enol tautomerization has previously been detected in involutin as well as in structurally related compounds, including chamonixin [27,28]. Based on their extensive NMR analyses, Kälvö and co-workers noted that these types of structures, with the inherent possibility of keto-enol tautomerization, impose various levels of difficulty during the NMR analysis and that the exact behavior of the compounds during analysis probably depends on factors such as solvent, pH, and temperature [28]. The resonances at 7.05 and 6.74 ppm in the NMR spectra of involutin have not been assigned since both resonances were not integer multiples of other resonances, i.e., they may relate to other molecules or impurities in the solution.

### 3.5. Production of Iron(III)-Reducing Metabolites over Time

**Figure 5:**
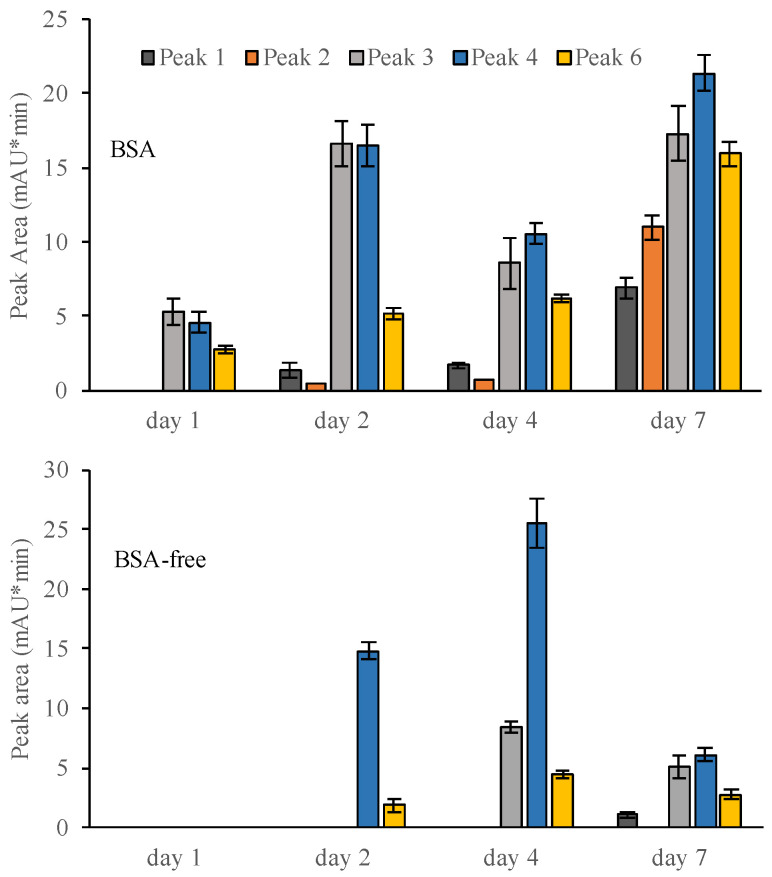
Secretion of iron(III)-reducing metabolites by *P. involutus* incubated for 1, 2, 4 and 7 days on medium containing BSA (top panel) or BSA-free medium (bottom panel). The peak area of five iron(III)-reducing metabolites are shown. These metabolites were separated using HPLC (λ = 254 nm). These metabolites were further characterized by mass spectrometry (Figure 4). Bars indicate ± standard deviations (*n* = 3). In all samples, equal volumes of culture filtrates were extracted.

The amounts of the five iron(III)-reducing metabolites that were present in the culture filtrates during incubation days 1, 2, 4 and 7 were estimated using HPLC-UV (Figure 5). Involutin was detected in the BSA medium from day 2, and its concentration increased on days 4 and 7. Hence, involutin was detected in the media shortly before the detection of the induced •OH production (c.f. Figure 1). The peak corresponding to involutin was not detected in any of the BSA-free samples. The remaining four iron(III)-reducing metabolites were produced by the fungus in both BSA and BSA-free media, but at different concentrations that also varied during the incubation period (Figure 5).

## 4. Discussion

**Figure 6:**
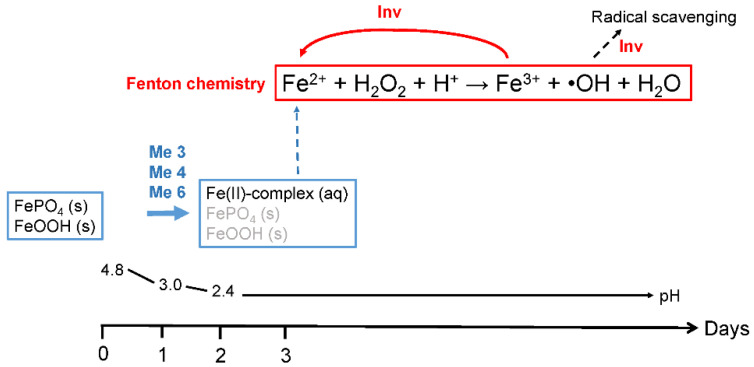
A schematic representation of the speciation of extracellular iron and production of iron(III)-reducing metabolites by *P. involutus* when grown for 7 days on BSA-containing medium. Initially, the iron is present as solid iron(III) phosphate and oxide complexes. These complexes are rapidly dissolved by not yet fully characterized iron(III)-reducing metabolites (denoted Me3, Me4 and Me6, corresponding to peaks 3, 4 and 6 in Table 3).The reduction results in the formation of soluble iron(II) complexes that are the main iron species present in the medium from day 2. Fenton chemistry is triggered on day 3, at a time when involutin (Inv) is detected in the medium. We propose that Inv is involved in reducing and recycling the iron(III) formed in the Fenton reaction. Inv can also be involved in scavenging the •OH radicals formed in the Fenton reaction and thereby protect the fungal hyphae from high levels of damaging radicals. See text for further details.

In soils, iron is mainly found in solid states, as Fe(hydr)oxides and in complexes with organic matter that can contain both iron(II) and iron(III) [24]. To initiate the Fenton reaction, *P. involutus* needs a mechanism to dissolve and reduce the iron(III) compounds/complexes. Previous work suggested that extracellular metabolites have an important role in the reduction of iron(III). Involutin was identified as the dominating iron(III)-reducing metabolite secreted by *P. involutus* during Fenton-based decomposition of a maize compost extract [15]. In the current work, we have examined in more detail the speciation of extracellular iron and the production of iron(III)-reductants when the fungus was growing on a protein medium where the Fenton reaction could be timely controlled and followed using a specific probe for detecting the production of •OH [18]. Our time-course data provided new insights into the mechanisms for solubilization and reduction of iron(III) complexes and the role of involutin in the Fenton reaction. The data are summarized in Figure 6.

Based on the XAS analysis, it is clear that iron in the media, added in the form of iron(III) chloride, is transformed and present as iron(III) phosphate and (hydr)oxide compounds prior to the growth of *P. involutus*. These forms of iron both have low solubilities in aqueous media. Within 24 h of growth, the vast majority of these Fe(III) species have been reduced to more water-soluble iron(II) complexes. The XAS results indicate that iron(II) in these complexes is coordinated to organic ligand(s), but presently the structure and composition of these ligands are unknown. Involutin was not detected in the medium within this timeframe. Hence, it is unlikely that involutin is responsible for the initial dramatic reduction of the insoluble iron(III) compounds. Instead, three other iron(III)-reducing metabolites were found in the medium at day 1, and one, or several of them, are probably involved in the reductive dissolution of the iron(III) phosphate and (hydr)oxide compounds. Notably, these metabolites were also secreted by the fungus when grown in the medium without BSA, suggesting that their production is stimulated by different queues than those triggering the production of involutin. We speculate that the production of the three iron(III)-reductants present at day one were induced by the presence of the insoluble iron(III) phosphate and (hydr)oxide compounds. It follows that these metabolites promote the release of phosphate associated with the insoluble iron(III) compounds through reductive dissolution. In soils, a major fraction of plant growth-limiting nutrients, including phosphorus, are associated with iron(III) oxide surfaces [29], and several studies have shown that *P. involutus* can access mineral bound P [30,31], but the precise mechanism(s) are still debated. However, studies in other microorganisms have shown that mineral-associated P can be released via reductive dissolution [32]. Our data suggest that *P. involutus* has the capacity to mobilize phosphorus from iron(III) minerals by secreting iron(III)-reducing metabolites.

Within the first 24 h of incubation of *P. involutus* on the BSA medium, the pH of the medium drops from 4.8 to 3 [18]. Under these acidic conditions, the re-oxidation of iron(II) by oxygen is slow [33,34], allowing the iron(II) complexes to accumulate in the growth medium. After 48 h of incubation, the soluble iron(II) complexes were the main iron species, and most likely, they serve as iron(II) reactants in the Fenton reaction initiated on day 3. The reaction mechanism by which the other Fenton reactant H_2_O_2_ is produced is currently still unclear. One possible reaction pathway is through autooxidation of iron(II) species and the concomitant reduction of O_2_, but as already mentioned at pH values <3.0, this reaction is very slow [33,34]. Another alternative is displayed by brown-rot fungi, which produce iron(III)-reducing metabolites, including the hydroquinone 2,5-dimethoxyhydroquinone (2,5-DMHQ), that are capable of generating both Fe^2+^ and H_2_O_2_ [13,35]. Studies have shown that 2,5-DMHQ can be oxidized to a semiquinone by the activity of an extracellular laccase, and the hydroquinone and semiquinone can together initiate a complete Fenton reaction system [36]. In vitro experiments showed that involutin produces •OH only in the presence of H_2_O_2_ [15], suggesting that this metabolite cannot reduce O_2_ to H_2_O_2_. Another possibility is that one or several of the four iron(III)-reducing metabolites present in the BSA medium at day three are able to reduce O_2_ to H_2_O_2_. Alternatively, H_2_O_2_ could also be generated by the activity of specific oxidases [37]. *P. involutus* express genes encoding such enzymes during SOM oxidation [11].

The Fenton reaction between Fe^2+^ and H_2_O_2_ re-oxidizes iron to Fe^3+^. In our experiment, this form of re-oxidized iron(III) is not known. However, no iron(III) species were detected by the XAS analysis, which suggests that the steady-state concentration of iron(III) was low. Involutin could be acting on the iron(III) species that are formed by the reaction of Fe^2+^ and H_2_O_2_, recycling the Fe^3+^ that is produced during the Fenton reaction. In brown rot fungi, the recycling of the Fe^3+^ Fenton reaction products back to Fe^2+^ is carried out by hydroquinones, including 2,5-DMHQ, produced by brown rot saprotrophs [38]. During the reaction, 2,5-DMHQ is oxidized to the corresponding quinone that is continuously reduced to the hydroquinone, allowing the redox cycle to continue [35]. It is not clear how the fungi reduce the produced quinones, but experiments suggest that the reaction involves the action of a quinone reductase enzyme present in the mycelium [39]. Maybe involutin is involved in a similar redox cycle as the hydroquinones/quinones in brown rot fungi. If so, it should be possible to detect both the reactive (reduced form) and oxidized form of involutin during the Fenton-based decomposition of proteins in *P. involutus*. Involutin could also be secreted by *P. involutus* in response to increasing •OH concentrations. In this case, the aromatic moieties of the involutin molecule could serve to scavenge excess •OH, as has been suggested to be the case for the iron(III)-reducing metabolite variegatic acid in *Serpula lacrymans* [40].

*P. involutus* belongs to the Boletales, which are nested within a paraphyletic assemblage of brown rot wood-decaying fungi, including *S. lacrymans* [7]. Brown rot fungi in the Boletales, but also in two other divergent lineages (Polyporales and Gloeophyllales), use a chelator-mediated Fenton (CMF) system to decay lignocellulose [41]. According to this model, fungal hyphae growing in the plant lumen produce oxalic acid that binds to and solubilizes iron(III) from insoluble iron(III) (hydr)oxides and decreases the pH near the fungal hyphae. The dissolution process is favored at the low pH value (~2) within the lumen area. The iron(III)- oxalate complex diffuses into the cell wall and encounter a significantly higher pH (~4.5) than in the lumen. At this pH, the secreted iron(III)-reducing compounds sequester the Fe^3+^ from the iron(III)-oxalate complex and reduce it to Fe^2+^ that subsequently can generate •OH via the Fenton reaction. *P. involutus* has the capacity to produce significant amounts of oxalic acid, and it has been shown to contribute to the dissolution of calcium bearing minerals at the hyphal-mineral interface [42,43]. However, the data presented in this paper do not support the involvement of an oxalate-driven CMF mechanism for Fenton-based decomposition of proteins by *P. involutus*. The XAS data showed that the solid iron(III) phosphate and (hydr)oxide compounds were dissolved by reductive dissolution and not by a nonreductive ligand-promoted mechanism. Furthermore, the pH value in the medium is ~2 on day three when the Fenton reaction is initiated [18]. Experiments with oxalic acids and iron(III)-reducing metabolites from brown rot fungi have shown that under such acidic conditions, the iron(III)-oxalate complexes are very strong, and the susceptibility to reduction is low [44].

Several iron(III)-reducing metabolites have been identified in *S. lacrymans*, including variegatic acid and 2,5 DMHQ, and their importance in the CMF mechanism associated with brown rot decay is disputed [38,45]. 2,5-DMHQ has so far not been detected in *P. involutus*, neither in the present study nor in previous experiments [15]. Variegatic acid is, like involution, a compound derived from atromentin, but it was not produced by *P. involutus* in the present study. Moreover, previous work has shown that variegatic acid and involutin are regulated by different mechanisms. Analysis of the promotor motifs of the gene clusters encoding atromentin synthetases in *P. involutus* and *S. lacrymans* revealed significant differences [46], which suggest that the metabolites produced by these enzymes are regulated by different signals in the two fungi. Dissimilar regulation is also supported by experiments showing that bacteria can significantly induce the production of atromentin-derived secondary metabolites in *S. lacrymans,* but not in *P. involutus* [46,47]. Collectively, the presented experimental results suggest major differences in the mechanisms underlying the Fenton chemistry in brown rot saprotrophs and ECM symbionts with regard to the role of oxalic acid and the regulation of iron(III)-reducing metabolites.

## Figures and Tables

**Table 1:** Shell-by-shell extended X-ray absorption fine structure (EXAFS) fit results. Samples were collected daily (from day 0 to day 7) from culture filtrates of *P. involutus* growing on BSA-containing media ^1^.

	Path	R (Å)	CN	σ^2^ (Å^2^)	ΔE_0_ (eV)
Day 7	Fe–O	2.12	3.2	0.0031	−9.1
Day 6	Fe–O	2.09	4.1	0.0069	−11.6
Day 5	Fe–O	2.10	4.6	0.0071	−10.7
Day 4	Fe–O	2.08	3.8	0.0050	−11.8
Day 3	Fe–O	2.08	3.9	0.0068	−9.5
Day 2	Fe–O	2.09	3.8	0.0067	−5.8
Day 1	Fe–O	1.99	5.6	0.0110	−11.8
	Fe–P	3.15	3.9	0.0085	
	Fe–Fe	3.10	3.9	0.0100	
Day 0	Fe–O	1.99	5.9	0.0088	−10.6
	Fe–P	3.19	3.5	0.0085	
	Fe-Fe	3.11	2.8	0.0100	

^1^ CN, coordination numbers; R(Å), interatomic distances; σ^2^ (Å^2^), Debye-Waller factor; ΔE_0_ (eV), threshold energy shifts. The amplitude reduction factor $\left(S_{0}^{2}\right)$ was set to 1.00.

**Table 2:** High-resolution electrospray ionization-mass spectrometry (HRESIMS) data of the isolated iron(III)-reducing metabolites from *P. involutus*. Peaks were collected using HPLC and assigned, as shown in Figure 2A. The mass spectra were acquired in negative mode. Elemental composition using the ring double bond (RDB) equivalent and the deviation of the observed mass from the theoretical mass (delta mma) for predicted chemical compositions were considered to predict the chemical formulas ^1^.

	RT (min)	MolecularIons (*m/z*)	Daughter Ions MS/MS (*m/z*)	RDB	Delta MMA	Predicted Formulas Molecular Ions
Peak 1	5.6	153.0181	109.0278, 153.0181	5.5	−0.365	C_7_H_6_O_4_
Peak 2	7.3	313.0716	135.0435, 149.0226, 163.0389, 295.2282, 313.0716	11.5	0.865	C_17_H_13_O_6_ (involutin)
Peak 3	7.8	327.0529	140.9846, 188.0342, 235.9256, 285.1706, 327.0529	12.5	0.881	C_17_H_12_O_7_
Peak 4	9.0	403.2330	201.1121, 403.2330	3.5	0.416	C_20_H_36_O_8_
Peak 6	16.2	333.0615	267.1600, 299.0557, 333.0615	10.5	1.276	C_16_H_14_O_8_

^1^ RDB is the measure of the degree of unsaturation in a molecule.

**Table 3:** Assignment of the peaks in ^1^H NMR spectra (at 500 MHz) of HPLC-purified involutin in the region of 6.5 to 7.9 ppm. The multiplicity and integrals are relative to the most stable involutin form and a tautomeric form (enol). Multiplicity: singlet (s), doublet (d) and distorted doublet (d *). The NMR spectra are shown in Figure S3, along with the carbon number assignment (C. No) on the molecular structure.

Chemical Shift (ppm)	Peak Multiplicity	Peak Integration (Number of Protons)	C. No
**Involutin**			
6.58	d	0.85	3′
6.68	s	0.84	6′
6.71	d	1.12	2′
6.76	d	2.1	8,10
7.74	d	2	7,11
**Involutin enol form**			
6.82	d	2.07	8,10
6.99	d	0.96	3′
7.26	d *	2.01	7,11
7.32	d	1.17	2′
7.89	d	1.01	6′

## Data Availability

The data presented in this study are available from the corresponding author upon reasonable request.

## Supplementary Materials

The following are available online at https://www.mdpi.com/2076-2607/9/1/35/s1, Figure S1: XANES spectra and the corresponding first-derivative of culture filtrate samples from the BSA growth medium. Figure S2: Iron-reducing metabolites in BSA culture filtrates and in Fries medium (control). Figure S3: Isolation of involutin from *P. involutus* for NMR analysis. Figure S4:^1^H NMR spectrum of HPLC-purified involutin.
