# Supplementary material for: Secretion of Iron(III)-Reducing Metabolites during Protein Acquisition by the Ectomycorrhizal Fungus Paxillus involutus
Source: Microorganisms. 2020 Dec 24;9(1):35. doi: 10.3390/microorganisms9010035 (PMC7824621; doi:10.3390/microorganisms9010035)

Figure S1. Pre-edge peak (A), X-ray absorption near-edge structure (XANES) (B) and first-derivative of XANES spectra (C) of culture filtrates collected daily from the growth of *P. involutus* cultures on a Fries medium containing ammonia and BSA as the nitrogen source. Blue dashed lines serve to distinguish changes to the peaks.

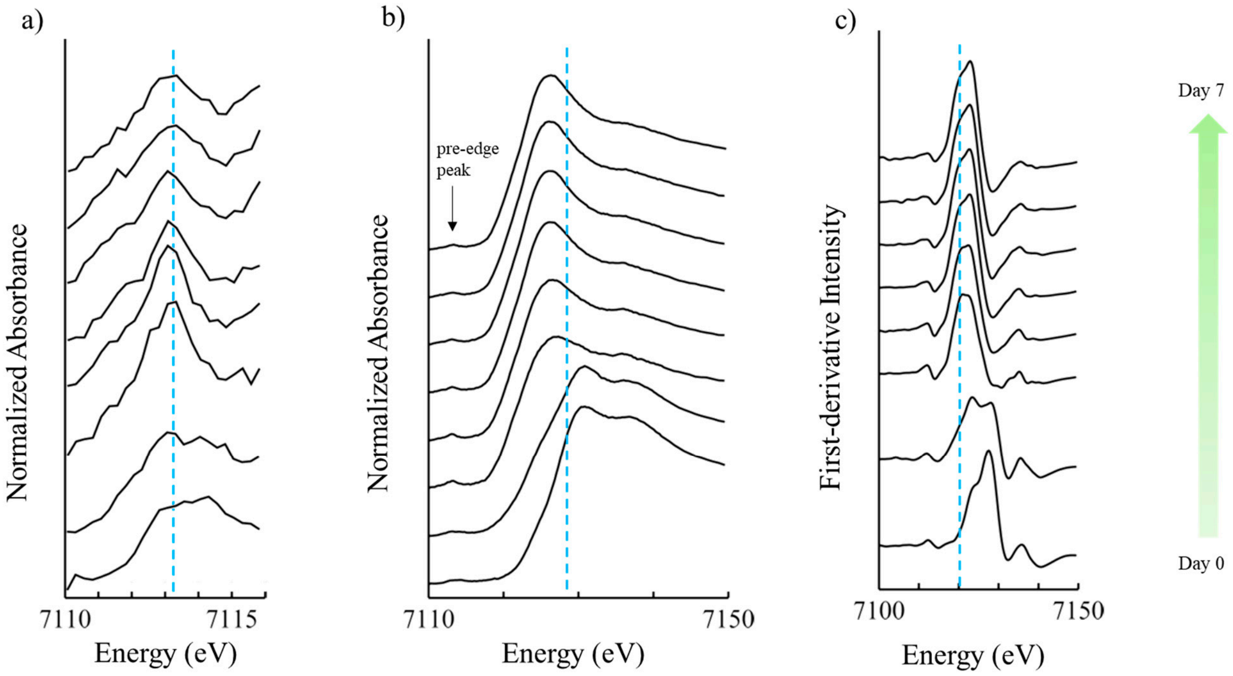

Figure S2. Iron-reducing metabolites in BSA culture filtrates and in Fries medium (control). The metabolites were extracted using equal volumes of ethyl acetate and separated using HPLC with a VWD-detector (extracted at λ=254 nm). "10_PAI Fries BSA" is the chromatogram of an extract of a BSA culture filtrate after 7 days of incubation; "Fries BSA CONTROL" is an extract of a Fries medium with BSA; and "Fries CONTROL" is a Fries medium without BSA added. Peaks with iron-reducing activities are designated 1-6 as in Fig. 4a (the retention times are different due to different chromatographic conditions). The peak assigned as involutin is indicated by an arrow. Note that peak 5 is present in the Fries medium and was not further characterized.

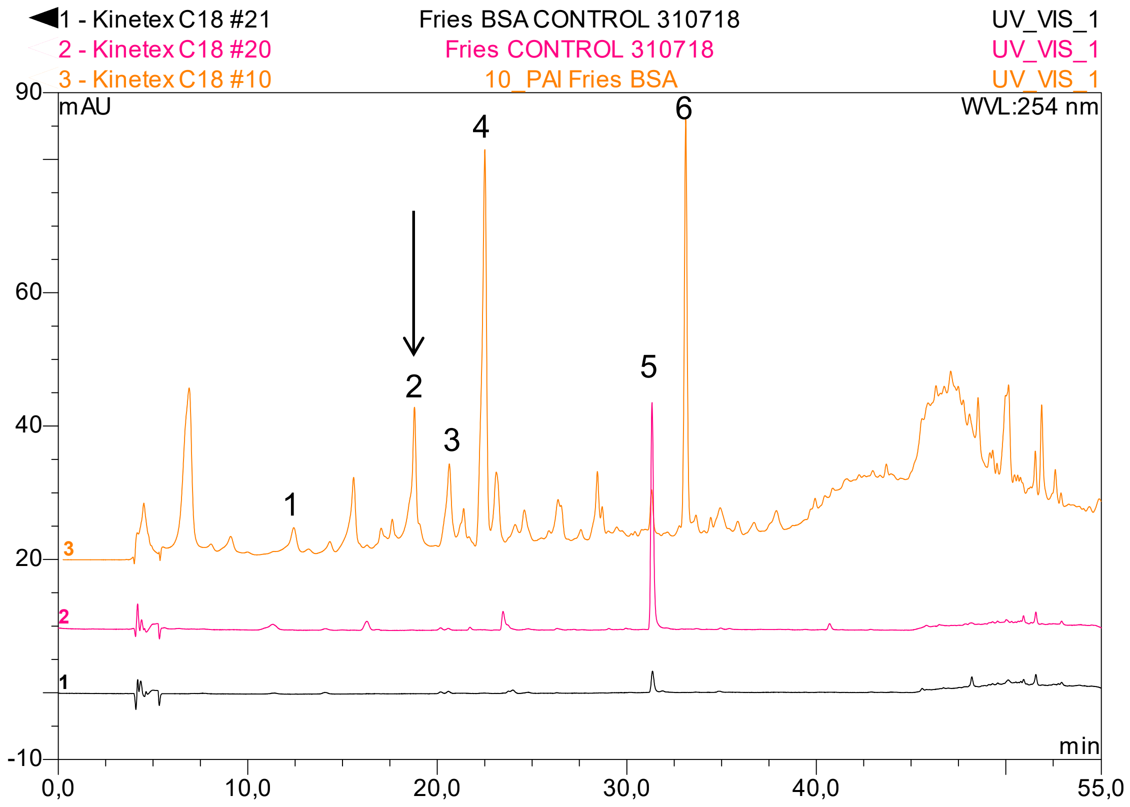

Figure S3. Isolation of involutin from *P. involutus* for NMR analysis. The fungus was grown in a malt extract liquid medium for 2 months and metabolites were extracted from the culture filtrates using ethyl acetate. (a) Analysis of the ethyl extract using HPLC with a VWD-detector (extracted at λ=254 nm) and the conditions used in Fig. S1. The peak assigned as involutin is indicated (arrow). (b) Re-chromatography of the "involutin" peak. The gradient is slightly different than that used in (a). (c) Mass spectrum of the "involutin" peak showing ions with *m/z* values corresponding to the molecular ions ([M-H]⁻) of involutin.

(A)

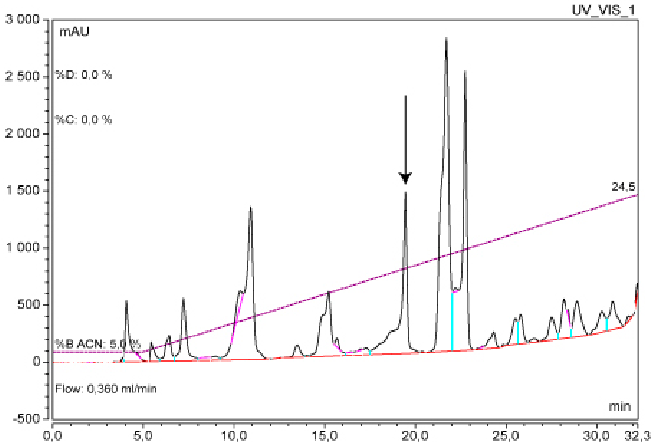

(B)

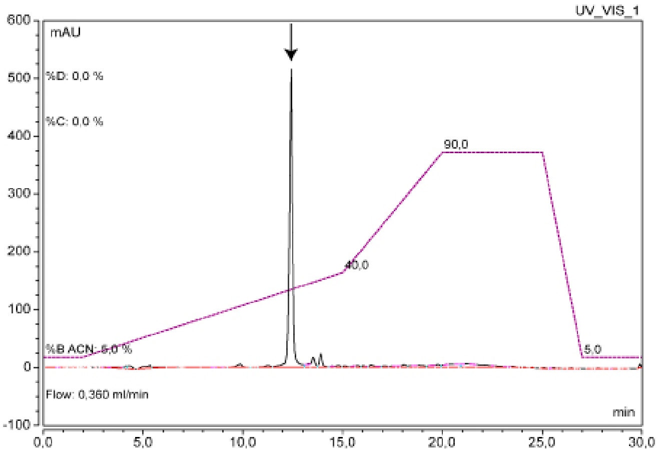

(C)

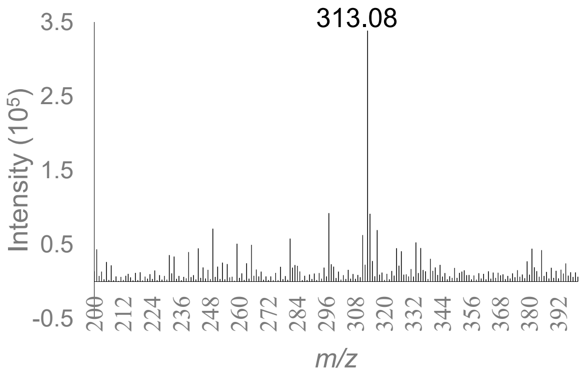

Figure S4. ^{1}H NMR spectrum of HPLC-purified involutin (A). A zoom of the ^{1}H NMR spectrum in the region 6.5-7.9 ppm shows the integrals relative to the most stable involutin form (B) and the integrals relative to the other tautomeric form (C). The red lines/numbers represent the integrator trace. The blue numbers represent the proton resonances of the chemical structure (Inset).

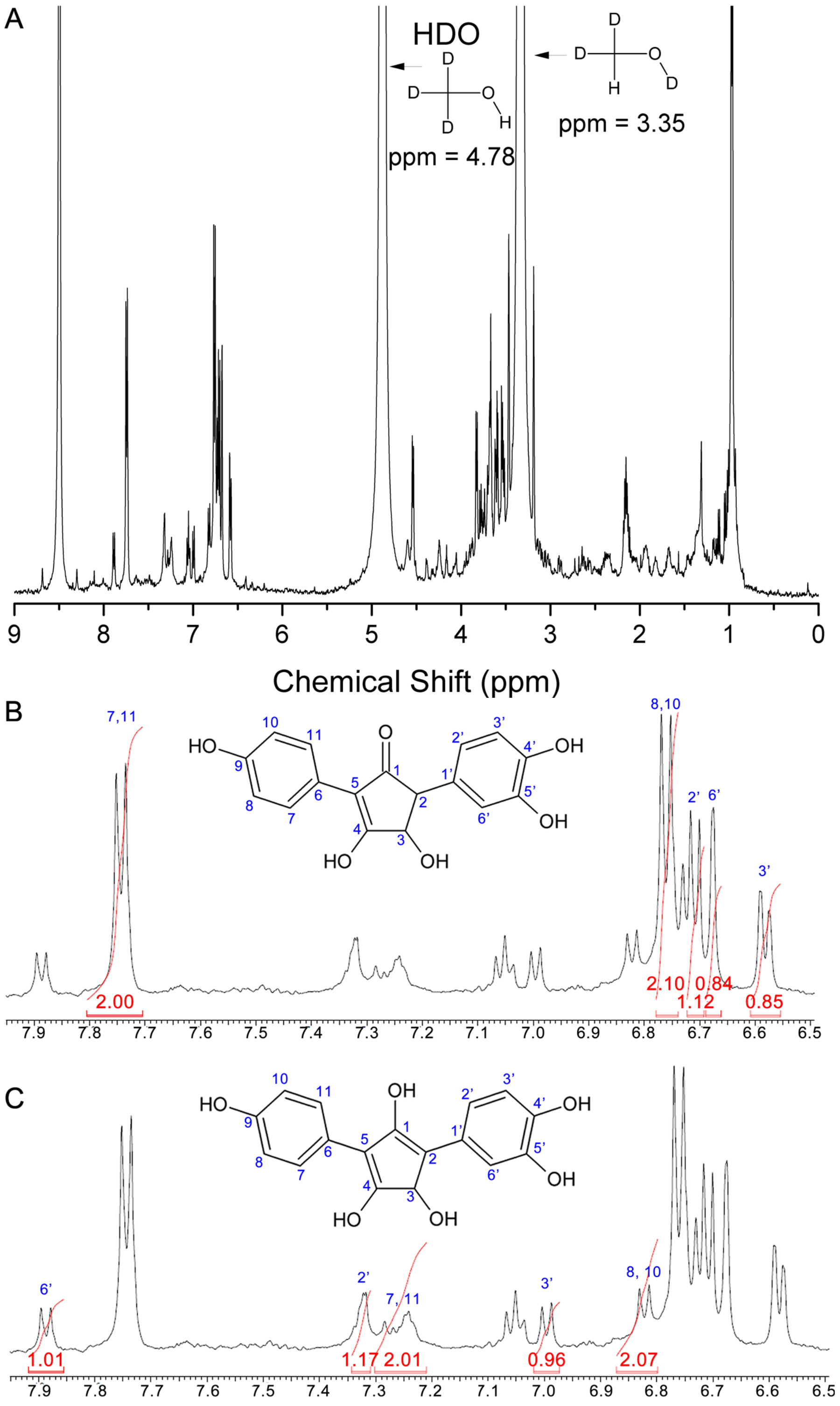

Supplement: Supplementary file 1 [file microorganisms-09-00035-s001.pdf]
